# Genomic diversity of *Ligularia* revealed by complete plastid genomes and nuclear ribosomal DNAs from 16 collections in Korean Peninsula and Mt. Hallasan

**DOI:** 10.1371/journal.pone.0343215

**Published:** 2026-02-19

**Authors:** Ju-Young Ahn, Seon Heui Jeong, Jong-Soo Kang, Jee Young Park, Yeon Jeong Kim, Yun Sun Lee, Jae Young Ko, Youn Gi Moon, Jung Hwa Kang, Tae-Jin Yang

**Affiliations:** 1 Department of Agriculture, Forestry and Bioresources, College of Agriculture & Life Sciences, Seoul National University, Seoul, Republic of Korea; 2 Department of Forest Resources, College of Forest and Environmental Sciences, Kangwon National University, Chuncheon, Republic of Korea; 3 Plant Genomics & Breeding Institute, Seoul National University, Seoul, Republic of Korea; 4 BK21 Agriculture-forestry Bioresource Convergence Center, Seoul National University, Seoul, Republic of Korea; 5 Crop Biotechnology Institute, Institutes of Green-bioScience and Technology, Seoul National University, Pyeongchang, Republic of Korea; 6 Department of International Agricultural Technology, Seoul National University, Pyeongchang, Republic of Korea; 7 Gangwon State Agricultural Research & Extension Services, Wild Vegetable Research Institute, Pyeongchang-gun, Gangwon State, Republic of Korea; 8 Hantaek Botanical Garden, Yongin, Republic of Korea; 9 Research Institute of Agriculture and Life Sciences, Seoul National University, Seoul, Republic of Korea; G. B. Pant Institute of Himalayan Environment & Development, INDIA

## Abstract

Plastid genome (plastome) and nuclear ribosomal DNA (nrDNA) sequences were assembled from 16 *Ligularia* accessions, including *L. fischeri*, *L. fischeri* var. *spiciformis*, and *L. stenocephala*. The assembled lengths ranged from 150,889 bp to 151,173 bp, while the 45S nrDNA transcription units varied from 5,846 bp to 5,848 bp. Comparative analyses revealed 0–106 SNPs and 0–60 InDels among the plastomes, and 1–50 SNPs and 0–4 InDels within the 45S nrDNA regions. Although the three taxa are morphologically distinct, phylogenetic analysis based on plastome and nrDNA sequences failed to differentiate them, with lineages from each taxon intermixing without clear separation. The incongruence between maternally inherited plastome- and biparentally inherited nrDNA-based phylogenies suggests frequent hybridization events, though incomplete lineage sorting may also contribute. This pattern of genetic admixture was also observed in accessions collected from various farms, implying that both natural and artificial hybridization have contributed to the current genetic structure. Samples from Mt. Hallasan showed broad genetic diversity, encompassing nearly all major variants found across the Korean Peninsula. Molecular markers were developed based on plastome variations and serve as grouping criteria. The genomic resources and markers generated in this study provide valuable tools for future molecular breeding and evolutionary research in *Ligularia*.

## Introduction

The genus *Ligularia* Cass., belonging to the Asteraceae family, is a perennial plant mainly distributed in Eurasia, with numerous species found in the Qinghai-Tibet Plateau of China [1,2]. This genus is one of the largest and most diversified genera in the Asteraceae, with 6 sections, 11 series, and 129 species [3–6]. According to previous studies, natural interspecific hybridization and outcrossing are frequent among morphologically distinct plant species within the genus *Ligularia*, which renders their taxonomic classification and identification difficult [4,7–9]. This phenomenon is also common in other Asteraceae plants, such as *Ambrosia artemisiifolia*, *Flourensia cernua*, and *Crepis sancta* [10–12]. This widespread tendency for interbreeding poses challenges for agricultural breeding programs. In South Korea, nine *Ligularia* species have been recorded [4]. Among them, *L. fischeri* and *L. stenocephala* are well-known edible herbs that have also gained popularity in the herbal medicine market [13].

*Ligularia fischeri* is distributed across East Asia and predominantly found in the eastern regions of South Korea, typically in the moist, shaded alpine environments at altitudes above 600 meters [14]. In Korea, the antioxidant properties of *L. fischeri* leaves have been utilized to treat various conditions, including hepatic insufficiency, rheumatoid arthritis, scarlet fever, and jaundice [15–18]. *Ligularia stenocephala* is found in Korea, Taiwan, Japan and has been used for its anti-diabetic, antioxidant, anti-ulcer, and hepatoprotective effects [19–22].

*Ligularia fischeri* and *L. stenocephala* can be distinguished by several morphological traits. Basal leaves differ between the two species, with *L. fischeri* bearing reniform to ovate-cordate blades, and *L. stenocephala* having cordate-hastate or reniform-hastate blades. Bracts in *L. fischeri* vary by position, ranging from ovate to lanceolate, while those of *L. stenocephala* are consistently lanceolate to linear-lanceolate. Floral differences include more ray florets and abundant tubular florets in *L. fischeri*, in contrast to fewer florets in *L. stenocephala*. Finally, the pappus of *L. fischeri* is typically brown to purplish, whereas *L. stenocephala* has a white or occasionally brown pappus [23]. *Ligularia fischeri* var. *spiciformis* is distinguished from *L. fischeri* by the presence of silvery hairs on the abaxial leaf surface, with the latter having green and hairless undersides [24].

Mt. Hallasan in Jeju Island, the highest mountain in South Korea, provides a unique ecological setting with distinct altitudinal and climatic gradients [25,26]. Such conditions may foster elevated levels of genetic diversity and act as a reservoir for native *Ligularia* populations. In addition, a recent comparative analysis of complete chloroplast genomes from six *Ligularia* species demonstrated that plastome data can effectively resolve phylogenetic relationships and function as a powerful super-barcode for species identification [27]. Recent advances in plastome and phylogenomic analyses have proven effective for resolving species boundaries and evolutionary relationships in angiosperms [28–30]. Nevertheless, comprehensive analyses remain limited for the genus *Ligularia*, and little is known about how such environments contribute to genetic structure within and between species.

In this study, we aimed to investigate the genetic variation and evolutionary relationships among 16 *Ligularia* samples collected across South Korea, including those from Mt. Hallasan. Specifically, we assembled the plastid genome (plastome) and 45S nuclear ribosomal DNA (nrDNA) sequences to explore sequence diversity, interspecific hybridization, and phylogenetic incongruence. Using these data, we developed molecular markers from single nucleotide polymorphisms (SNPs) and insertions and deletions (InDels) to assess genetic diversity and population structure. Our results indicate that L. fischeri, L. fischeri var. spiciformis, and L. stenocephala may belong to a single species complex, highlighting the evolutionary and genetic complexity of these valuable herbal plants.

## Materials and methods

### Plant materials and genome sequencing

A total of 72 *Ligularia* samples were collected from various regions of South Korea and provided by Gangwon Agricultural Research and Extension Services Wild Vegetable Experiment Station and Hantaek Botanical Garden (S1 Table in S2 File). All field collections were conducted in accordance with national and municipal biodiversity and conservation regulations. No specific permits were required for the species used in this study. From this initial set, sixteen representative samples (Table 1) were selected for genomic analysis. This subset included five accessions from plants growing at altitudes of 1,400–1,950 meters on Mt. Hallasan (Table 1). Total genomic DNA was extracted from leaf tissue using the GeneAll PLANT SV MINI kit (Geneall Biotechnology Ltd., Seoul, South Korea). The integrity and quality of the extracted DNA were verified by agarose gel electrophoresis, and its concentration was measured using NanoDrop ND-1000 (NanoDrop Technologies, Inc., Wilmington, DE, USA). Sequencing and library preparation for all 16 samples were performed by Phyzen (PHYZEN CO., LTD., Seongnam, South Korea) on an Illumina Miseq platform (Illumina, San Diego, CA, USA). The sequencing depth of the chloroplast genome ranged from 21× to 81× (mean ≈ 46×), while the 45S nrDNA region showed 44 × –196 × coverage, indicating sufficient read depth for accurate assembly and analysis (S2 Table in S2 File).

**Table 1:** Information about 16 *Ligularia* samples used for next-generation sequencing.

Scientific name	Sample name	Sources (collection sites)	Accession number (cp/nrDNA)
*L. fischeri*	Lf_1	Hantaek botanical garden (Mt. Hallasan, Jeju-Island alt. 1400m)	PX608188/ PX631056
	Lf_2	Mt. Hallasan, Jeju-Island (alt. 1770m)	PX591403/ PX631055
	Lf_3	Mt. Hallasan, Jeju-Island (alt. 1810m)	PX591404/ PX631057
	Lf_4	Mt. Hallasan, Jeju-Island (alt. 1905m)	PX591405/ PX631058
	Lf_5	Mt. Hallasan, Jeju-Island (alt. 1950m)	PX591406/ PX631059
	Lf_6	Hantaek botanical garden (Inje, Gangwon State)	PX608189/ PX631068
	Lf_7	Hantaek botanical garden (Aewol-eup, Jeju-Island)	PX608192/ PX631069
*L. fischeri* var*. spiciformis*	Lf_19	Wild vegetable experiment station, GWARES (Taebaek-si, Gangwon State)	PX591407/ PX631064
	Lf_20	Hantaek botanical garden (Yongin-si, Gyeonggi-do)	PX608191/ PX631065
	Lf_21	Natural Product Research Center, KIST Gangneung Institute (Gangneung-si, Gangwon State)	PX591408/ PX631066
*L. stenocephala*	Ls_1	Wild vegetable experiment station, GWARES (Sinan-gun, Jeollanam-do)	PX591409/ PX631062
	Ls_2	Hantaek botanical garden (Yeosu-si, Jeollanam-do)	PX608190/ PX631060
Farm collection	Farm_1	Wild vegetable experiment station, GWARES (Hoengseong-gun, Gangwon State)	PX591399/ PX631061
	Farm_2	Wild vegetable experiment station, GWARES (Hwacheon-gun, Gangwon State)	PX591400/ PX631063
	Farm_3	Wild vegetable experiment station, GWARES (Pyeongchang-gun, Gangwon State)	PX591401/ PX631067
	Farm_4	Wild vegetable experiment station, GWARES (Ulleung-gun, Gyeongsangbuk-do)	PX591402/ PX631070

### Plastome and 45S nrDNA assembly and annotation

Plastome and 45S nrDNA sequences were assembled from low-coverage whole-genome sequencing (dnaLCW) method [31,32] using the CLC Assembly Cell (ver. 4.6 beta; CLC Inc., Aarhus Denmark). Briefly, raw paired-end reads were trimmed by CLC quality trim software with an offset of quality score value of 33. The trimmed reads were then assembled with an overlap distance ranging from 150 to 500 bp. The initial contigs were extracted using MUMmer [33] and then arranged by comparison to the reference plastome sequence of *Ligularia fischeri* Nakai (accession number: KT988070.1) [34]. The assembled plastomes were validated through manual curation of raw read mapping. The reference sequence for 45S nrDNA was derived from *Brassica oleracea* (accession number: MN401694.1) in the NCBI GenBank. In addition, two related species (*L. fischeri*, *L. stenocephala*) in the tribe Senecioneae, which were assembled using short read archive (SRA) data of NCBI GenBank (SRR13950043 and SRR13300173), were used as references to 45S nrDNA phylogeny. Gene annotation was conducted using GeSeq (https://chlorobox.mpimp-golm.mpg.de/geseq.html) [35], and circular maps of the plastome were generated using the OGDRAW program [36], and manually curated using the ARTEMIS annotation tool [37]. The 45S nrDNA region was annotated using a BLAST search, which included the 18S, 5.8S, 26S, and two internal transcribed spacer (ITS) sections.

### Comparative and phylogenetic analyses of plastomes and 45S nrDNA sequences

The assembled plastome sequences from 16 *Ligularia* samples were aligned and compared using the MAFFT 7.0 [38] to identify polymorphic sites. Multiple alignments included plastomes of nine related species, belonging to the tribe Senecioneae, such as *Ligularia fischeri* (KT988070.1), *L. stenocephala* (MT985379.1), *L. jaluensis* (MF539931.1), *L. intermedia* (MF539930.1), *L. mongolica* (MF539932.1), *L. hodgsonii* (MF539929.1), *L. veitchiana* (MF539933.1), *L. virgaurea* (MN783367.1), and *L. biceps* (OK448480.1). These sequences were downloaded from NCBI GenBank. The alignment of 45S nrDNA sequences of 16 *Ligularia* samples was also performed using MAFFT. *Farfugium japonicum* (MF929248.1), a member of the Asteraceae family, was included as an outgroup.

Based on aligned plastome and 45S nrDNA sequences, phylogenetic analyses were performed using randomized axelerated maximum likelihood (RAxML Version 8) [39] with 1000 bootstrap replicates and a substitution model of GTRGAMMA. Bootstrap values greater than 50% are shown above the branches.

### Development and validation of molecular markers for the genus *Ligularia*

Molecular markers were developed based on the polymorphic sites in plastome sequences of 16 *Ligularia* samples. Primers were designed using primer designing tool in Primer3 [40] and NCBI (Table 2). Polymerase chain reaction (PCR) amplification with InDel markers were performed in a final volume of 25 μL, containing 10 ng of template DNA, 10X Inclone Taq buffer, 10 mM dNTPs, 10 pmol of each primer, and 5 U/μL of Taq DNA polymerase (Inclone Biotech CO., Yongin, South Korea). PCR conditions of InDel markers were as follows: 5 min at 95°C, 35 cycles of 95°C for 20 sec, 56.1–57.7°C (according to primer Tm) for 30 sec, and 72°C for 20 sec; and the final extension at 72°C for 5 min. PCR amplicons were analyzed using 3% agarose gel and Inclone^TM^ Safe Gel stain. Then they were visualized under a UV Transilluminator and a gel documentation system. PCR reactions for high-resolution melting (HRM) analysis of SNP targets were performed in 20 μL final volume that is composed of 10 ng of template DNA, 2 μL of 10X Inclone Taq buffer, 0.4 μL of 10 mM dNTPs, 10 pmol of each primer, and 0.2 μL of Taq DNA polymerase and 1 μL SYTO 9 (Thermo Fisher, USA). PCR conditions were as follows; 5 mins at 95°C, 45 cycles of 20 sec at 95°C, 20 sec at 55.3–57.6°C, and 20 sec at 72°C. Then HRM analysis was conducted using LightCycler 96 (Roche Applied Science, Germany). HRM conditions were as follows; 1 min at 95°C, 1 min at 40°C, 5 sec at 60°C with temperature increasing up to 95°C, then decreasing to 40°C while fluorescence was acquired.

**Table 2:** Information of eight developed markers to determine genotypes of *Ligularia* species.

Marker type	Primer name	Region	Primer sequences (5’- 3’)	Tm(°C)
**INDEL**	Ligu_1	*rpoC1*	F: TGCTTGGTCTTCACTGGAAACT R: TTCATCCGGCTCAAGTAGGT	57.4
	Ligu_2	*trnG*	F: CTTTGGTTTCATTCGGCTCCT R: CGCCTTCTTCTAGAGGGATCA	57.7
	Ligu_3	*trn-psbD*	F: GGCTTGAGGTACCAAAAATTTCCA R: AGGAGACCAGAAATGAGAGTATTT	56.1
**SNP**	Ligu_4	*rps8*	F: GGCCGACTGATCCGTTTTA R: CGAAAACGTGAGAAAACATCG	55.6
	Ligu_5	*trnQ-rps16*	F: CATAACTAACAGAAATCGCAGGA R: GGATTGAATCTTTGGGAAGTTG	54.6
	Ligu_6	*psbI-trnC*	F: TCACGAATTCTCAGTCAACGA R: GCCGAAAAACTTCCACTCAC	56.4
	Ligu_7	*psbA-trnH*	F: AACTTCATAAAAGATTGGGAAAAGG R: TTTGGTTCGATTCGCGTCT	55.3
	Ligu_8	*rps16-intron*	F: TCATTTGTACTCATAACTCAAGTTCAA R: TGTCTCACGGATCCGAATAA	55.1

## Results

### Plastome and 45S nrDNA assembly of 16 *Ligularia* samples

**Fig 1:**
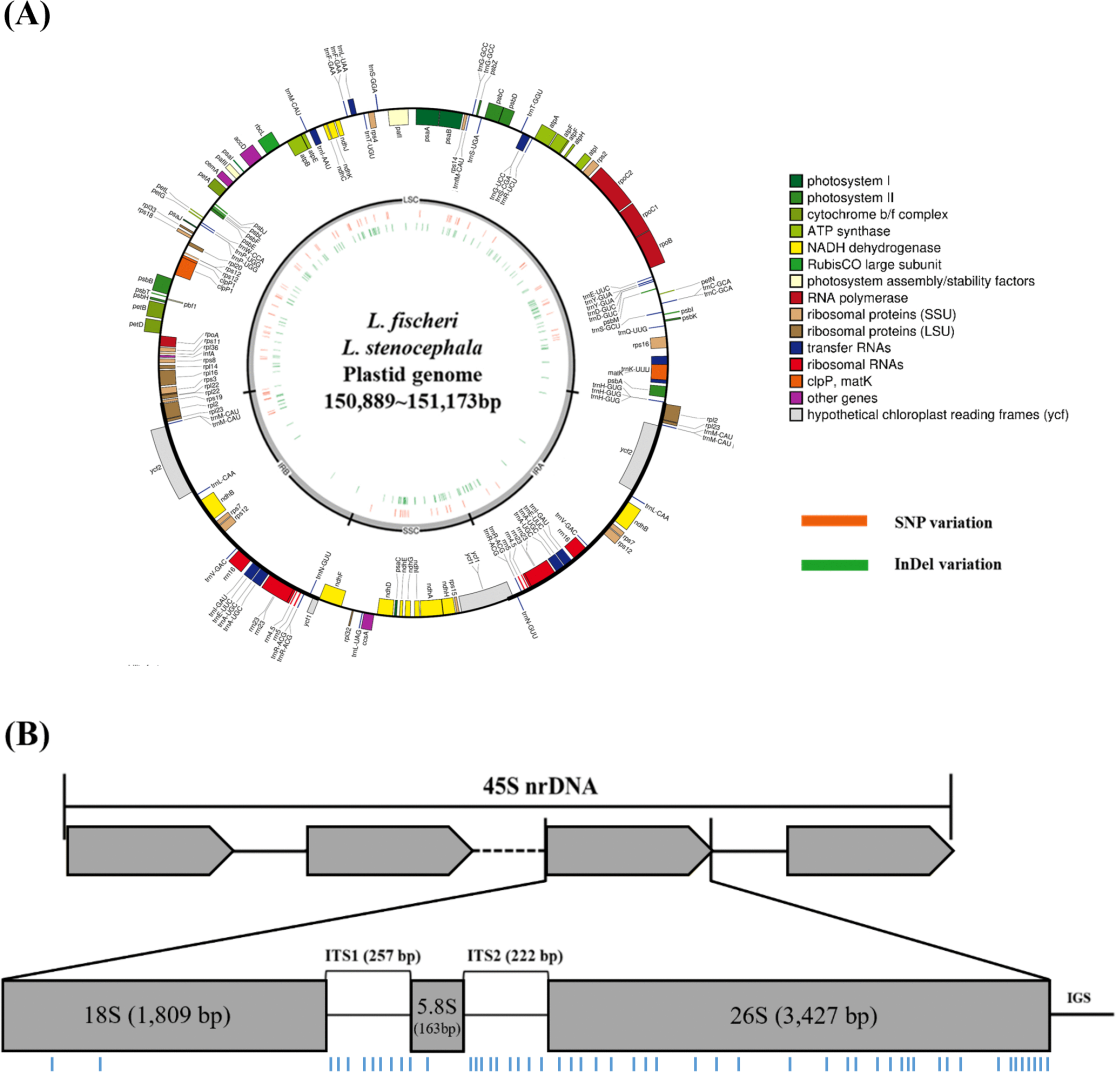
Plastid genome map and scheme of 45S nrDNA with sequence variations among 16 *Ligularia* samples. **(A)** Circular map was generated using the OGDRAW. SNPs and InDels are indicated in the inner circle. The outer and inner colored boxes represent transcribed clockwise and counterclockwise genes, respectively. The plastome is divided into a Large Single-Copy (LSC) region, Small Single-Copy (SSC) region, and two Inverted Repeat (IR) regions, which are labeled on the map. The color coding represents gene identification or function. **(B)** The upper boxes indicate the structure of tandemly arranged 45S nrDNA genes, and the lower boxes indicate the structure of the 45S nrDNA. The blue bars represent the positions of the variation sites based on comparison of 45S nrDNA sequences among 16 *Ligularia* samples.

Approximately 1 Gbp of high-quality NGS data was obtained from 16 *Ligularia* samples and used for the *de novo* assembly of plastomes and 45S nrDNA sequences (S2 Table in S2 File). The total lengths of the 16 assembled plastomes ranged from 150,889 bp to 151,173 bp, which were similar to the previously reported *L. fischeri* plastome [27]. These plastomes exhibited the typical circular quadripartite structure, including a small single-copy (SSC) region and a large single-copy (LSC) region, separated by two inverted repeat (IR) regions (Fig 1). The length of the LSC region ranged from 82,992 bp to 83,284 bp, the SSC region from 24,830 bp to 24,831 bp, and the IR regions from 18,224 bp to 18,249 bp (S2 Table in S2 File). The overall GC conte nt of the plastome sequences ranged from 37.45%–37.47% across all 16 samples, with the IR regions showing the highest GC content (41.22%–41.36%), followed by the SSC region (38.16% – 38.18%) and the LSC region (35.60–35.68%). All plastomes contained 87 protein-coding genes, 37 tRNA genes, and 8 rRNA genes (Fig 1A). There were no differences in the number of genes among the plastomes. The assembled 45S nrDNA transcription unit sequences of the 16 *Ligularia* samples ranged from 5,846 bp to 5,848 bp. Additionally, the assembled 18S nrDNA region was 1,809 bp in length, the ITS1 region was 257 bp, and the 5.8S was 163 bp. The ITS2 region ranged from 220 bp to 222 bp, and the 26S region was 3,427 bp (Fig 1B).

### Sequence variations among 16 *Ligularia* collections

Sequence alignments revealed variable regions in both plastome and 45S nrDNA sequences among the 16 *Ligularia* samples. Pairwise comparisons of 16 plastomes revealed 0–106 SNPs and 0–60 InDels (S3 Table in S2 File). The positions of SNPs and InDels were shown on the plastome map (Fig 1A). Compared to the IR region, relatively more SNPs and InDels are displayed in the LSC and SSC regions. Similarly, 1–50 SNPs and 0–4 InDels were identified among 16 45S nrDNA sequences (Fig 1B, S4 Table in S1 File). The 18S and 5.8S regions showed relatively fewer variations than the other subunits of the 45S nrDNA. A total of 52 heterozygous sites were detected in the 45S nrDNA sequences of the 16 samples (S5 Table in S2 File).

### Phylogenetic analyses of *Ligularia* samples based on the plastomes and 45S nrDNA sequences

**Fig 2:**
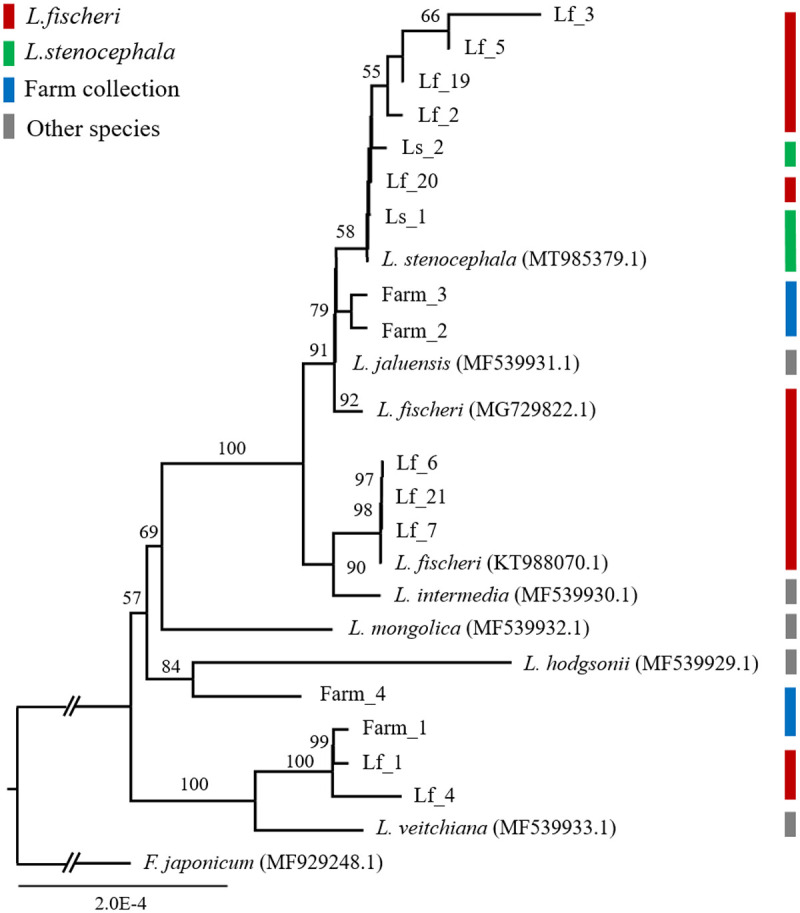
Maximum-likelihood tree based on the concatenated 78 plastid gene sequences of 16 *Ligularia* samples. A phylogenetic tree was constructed using the conserved coding sequences (CDSs) of genes shared among 16 newly sequenced and assembled samples and 6 closely related species. Phylogenetic analysis was conducted in RAxML [39] with 1000 bootstrap replicates under the GTRGAMMA substitution model. The GenBank accession numbers are shown in parentheses. *Farfugium japonicum*, a member of the Asteraceae family, was used as the outgroup. Bootstrap values greater than 50% are shown above the branches. An asterisk indicates samples from the Mt. Hallasan collections. Lf: *L. fischeri*, Ls: *L. stenocephala*.

To infer the phylogenetic relationships among the collected *Ligularia* species, 16 newly sequenced plastomes were analyzed along with 9 publicly available plastomes from the same genus, with *Farfugium japonicum* included as an outgroup (Fig 2). The plastome-based phylogenetic tree revealed that the 16 *Ligularia* samples were distributed across highly diverse lineages. One farm accession (Farm_1) and two *L. fischeri* samples (Lf_1 and Lf_4) formed a sister group with *L. veitchiana*, while one farm accession (Farm_4) clustered more closely with *L. hodgsonii*. In addition, three *L. fischeri* samples (Lf_6, Lf_7, and Lf_21) showed a close relationship with *L. intermedia*. Meanwhile, four *L. fischeri* samples (Lf_2, Lf_5, Lf_19 and Lf_20) and two *L. stenocephala samples* (Ls_1 and Ls_2) grouped with *L. stenocephala*, and two farm accession (Farm_2 and Farm_3) were most closely related to *L. jaluensis*. Among the *L. fischeri* samples, Lf_3 was found to be closely related to *L. biceps*. These results indicate that the collected *L. fischeri*, *L. stenocephala*, and *L. fischeri var*. *spiciformis* samples were not clearly separated from other *Ligularia* species based on plastome data.

**Fig 3:**
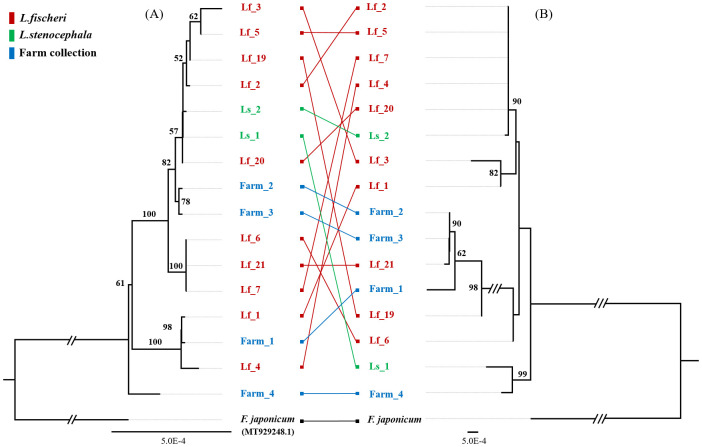
Maximum-likelihood phylogenetic trees of 16 *Ligularia* samples inferred from plastome and nrDNA sequences. The phylogenetic tree was inferred based on complete plastome and nrDNA sequences from 16 newly sequenced samples. Two phylogenetic analyses were conducted in RAxML [39] with 1000 bootstrap replicates under the GTRGAMMA substitution model. The outgroup is *Farfugium japonicum*, a member of the Asteraceae family. Bootstrap values greater than 50% are shown above the branches. (A) plastome-based phylogenetic tree. (B) nrDNA-based phylogenetic tree.

To complement this analysis, a phylogenetic tree was also constructed using the ITS1–5.8S–ITS2 region of 45S nuclear ribosomal DNA (nrDNA) from the same 16 *Ligularia* samples, with *F. japonicum* again used as an outgroup (Fig 3). Comparison of the plastome- and nrDNA-based phylogenies revealed topological inconsistencies among samples across the two datasets. In particular, *L. fischeri*, *L. fischeri* var. *spiciformis* and *L. stenocephala* could not be clearly distinguished in either tree, and the samples collected from the farms were also inconsistently separated. For example, Ls_1 was closely related to Ls_2 in the plastome tree but showed a closer relationship to Farm_4 in the nrDNA tree.

### Genetic diversity of *L. fischeri* along altitudinal gradients in the Mt. Hallasan

**Fig 4:**
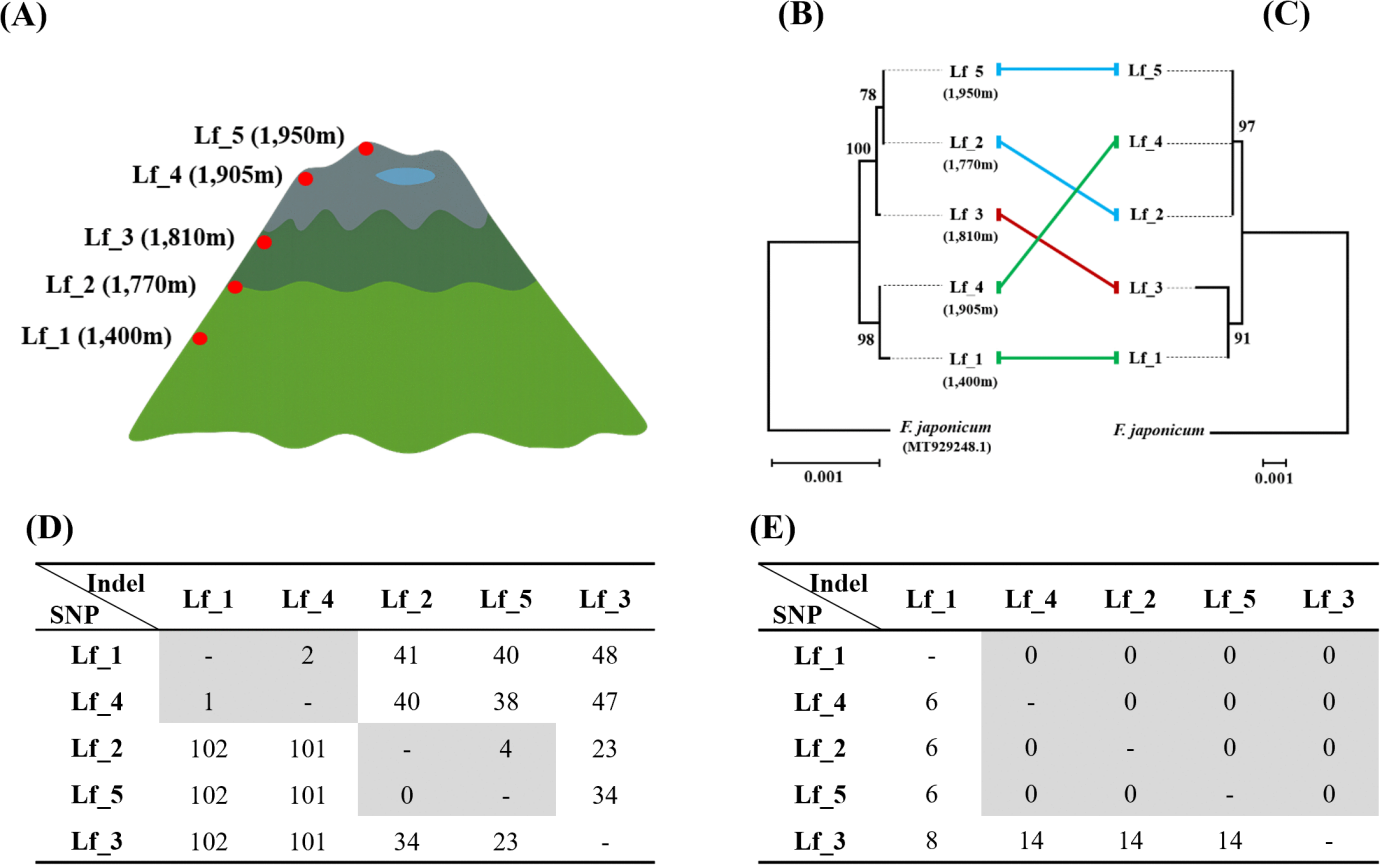
Maximum-likelihood phylogenetic trees of five Mt. Hallasan collection samples inferred from plastome and nrDNA sequences. The outgroup used in this study was *Farfugium japonicum*, a member of the Asteraceae family, and the 45S nrDNA was assembled using SRA data (SRX9729095). Bootstrap values greater than 50% are shown above the branches. **(A)** Illustration showing the locations of sample collection sites in the Mt. Hallasan region. **(B)** Plastome-based phylogenetic tree. (C) nrDNA-based phylogenetic tree. **(D)** Numbers of SNPs and InDels in plastomes among five Mt. Hallasan collections. **(E)** Numbers of SNPs and InDels in 45S nrDNA sequences among five Mt. Hallasan collections.

Five *L. fischeri* samples collected from altitudes between 1,400 and 1,950 m on Mt. Hallasan were analyzed for intraspecific genetic variation (Fig 4A). Alignment of plastome sequences revealed that Lf_1, collected at 1,400 m, differed by 102 SNPs from the other samples except Lf_4, which was collected at 1,905 m. The number of InDels between Lf_1 and others ranged from 40 to 48 (Fig 4D). In the plastome phylogeny, Lf_1 and Lf_4 formed a sister group to another clade containing Lf_2, Lf_3, and Lf_5. Within this clade, Lf_2 and Lf_5 formed a subclade, with Lf_3 as the sister clade to this subclade (Fig 4B).

Comparison of nrDNA sequences showed that, similar to the plastome, Lf_1 and Lf_3 showed the largest number of SNPs with a total of eight, and, except for Lf_3, no InDels were detected in any sample (Fig 4E). Unlike the plastome-based phylogeny, the nrDNA-based phylogeny showed that Lf_1 and Lf_3 formed one clade, while Lf_2, Lf_4, and Lf_5 formed another clade (Fig 4C). We observed a phylogenetic discordance in the placement of Lf_3 and Lf_4 between the plastome- and nrDNA-based phylogenies. A total of 45 heterozygous sites were observed in the 45S nrDNA sequences among the Mt. Hallasan samples (S5 Table in S2 File).

### Development of molecular markers based on plastome diversity

**Fig 5:**
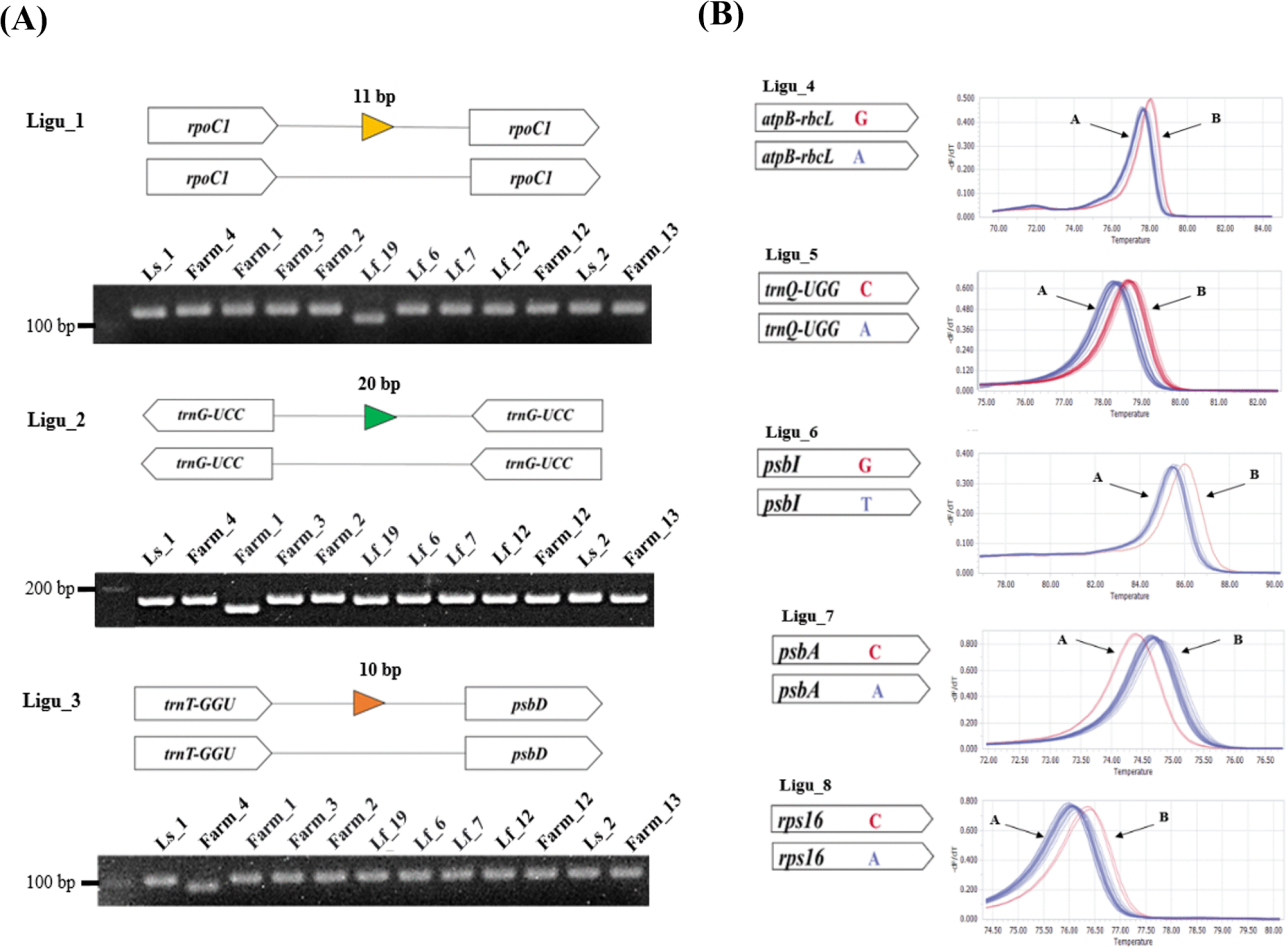
Scheme and results of three InDel markers and five SNP markers based on plastome sequences of 16 *Ligularia* samples. **(A)** Test results of 10 *Ligularia* samples using InDel markers. **(B)** HRM results using 5 SNP markers.

**Fig 6:**
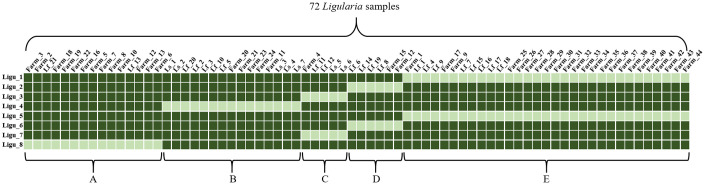
Results of marker tests with 72 extensive *Ligularia* samples. Five genotypes **(A, B, C, D,** and **E)** were distinguished using three InDel markers (Ligu_1, Ligu_2, Ligu_3) and five SNP markers (Ligu_4, Ligu_5, Ligu_6, Ligu_7, Ligu_8). Dark and light green colors indicate different genotypes.

To assess genetic diversity and identify genotypes, we developed PCR-based molecular markers using polymorphisms identified in the *Ligularia* plastomes through phylogenetic and mutation analysis. A total of three InDel markers and five SNP markers were developed and applied to 12 *Ligularia* samples (Lf_6, Lf_7, Lf_12, Lf_19, Ls_1–2, Farm_1–4, and Farm_12–13) (Fig 5A and Table 2). The Ligu_1 marker, which targets an 11 bp InDel in the *rpoC1* intron sequence, was able to distinguish only Lf_19. The Ligu_2 marker, targeting a 20 bp InDel in the *trnG* intron region, distinguished only Farm_1. Similarly, the Ligu_3 marker, targeting a 10 bp InDel in the *trnT*-*psbD* region, distinguished only Farm_4. Additionally, SNPs were identified in the plastome regions, including the *rps8* gene, *trnQ*-*rps16*, *psbI*-*trnC*, *psbA*-*trnH*, and *rps16* intron regions, with G/A, C/A, G/T, C/A, and C/A variations, respectively. Based on these, SNP markers were developed and validated in the same 12 samples (Fig 5B). The five markers (Ligu_4–8) were verified using HRM analysis, with each showing a distinct melting curve. The eight designed markers were applied to a comprehensive set of 72 *Ligularia* samples for genotyping analysis (Fig 6). The 72 samples were divided into five groups (A, B, C, D and E) based on the combination of the results from these eight markers, again showing that distinguishing *L. fischeri* and its related taxa is challenging with these molecular approaches.

## Discussion

### Genetic variation in the plastome and nrDNA among the *Ligularia* genus

A total of 16 *Ligularia* resources of *L. fischeri*, *L. fischeri* var. *spiciformis*, and *L. stenocephala* collected from various regions in South Korea were successfully sequenced and assembled with complete plastid genomes and 45S nrDNA (Fig 1). Complete plastid genomes have been successfully used as ‘super-barcodes’ for species identification in a recent study, including *L. intermedia*, *L. jaluensis*, *L. mongolica*, *L. hodgsonii*, *L. veitchiana*, and *L. fischeri* [27]. However, in our 16 samples, we found considerable variations between the plastome and nrDNA sequences (S2 and S3 Tables in S2 File). In particular, Lf_1 and Lf_2, which are both classified as *L. fischeri*, showed the largest difference with 100 SNPs. However, there was almost no sequence difference between Lf_2 (*L. fischeri*), Lf_20 (*L. fischeri* var. *spiciformis*), and Ls_2 (*L. stenocephala*), with only one SNP observed. This clearly indicates that it is difficult to distinguish species based on plastome-based phylogenetic trees (Fig 3A). In addition, the three *L. fischeri* var. *spiciformis* individuals (Lf_19–21) were not grouped into the same clade. These results provide evidence of active hybridization among the three species across different regions. Although the three species are morphologically distinguishable [23,24], these species are genetically difficult to differentiate.

To enhance the accuracy of sample identification, we conducted the assembly of nrDNA from all 16 *Ligularia* samples, generating a separate phylogenetic tree (Fig 3B). In angiosperms, plastid inheritance is thought to be predominantly maternal, with 20% of species potentially having biparental plastid inheritance [41]. In contrast, 45S nrDNA is inherited from both parents [42] and is used to study hybrids of various plant species [43,44]. Surprisingly, compared to the plastome-based phylogenetic tree (Fig 3A), the nrDNA-based phylogenetic tree exhibited remarkably distinct topological arrangements. In addition, the 45S nrDNA nucleotide type and mapping depth were also analyzed to confirm the possibility of hybridization. Sixteen *Ligularia* samples showed heterozygous regions at various positions (S5 Table in S2 File). Since the 45S region is generally conserved in plants, the presence of such heterozygous sites may serve as evidence supporting the possibility of hybridization between closely related species.

Such incongruence between plastome- and nrDNA-based phylogenies is commonly reported in many Asteraceae taxa and may arise from multiple evolutionary processes [45–47]. While hybridization and chloroplast capture are well-known causes [48], incomplete lineage sorting (ILS) is also a major factor contributing to conflicting gene trees, particularly in rapidly diversifying groups [49]. Similar patterns have been documented in related genera such as *Senecio* and *Saussurea* [46,50], where both hybridization and ILS jointly produce nuclear–plastid phylogenetic discordance. Therefore, the incongruence observed in our *Ligularia* samples may reflect not only frequent hybridization but also incomplete lineage sorting among recently diverged lineages.

### Possibility of interspecific hybridization within the *Ligularia* genus

Previous studies have indicated that interspecific hybridization occurs frequently within the genus *Ligularia* [51,52]. To further investigate this, we compared the plastome of the 16 samples analyzed in this study with all publicly available *Ligularia* plastomes (Fig 2). The resulting phylogenetic tree revealed notable relationships. Specifically, Lf_1 and Lf_4, both native to Mt. Hallasan, clustered together with farm-collected samples and *L. veitchiana*. In addition, Lf_3 grouped closely with *L. biceps*, while Farm_4 showed a close phylogenetic relationship with *L. hodgsonii*. According to Plants of the World Online (2025), *L. biceps* is native to Liaoning Province in northeastern China [53], *L. veitchiana* is native to central China, and *L. hodgsonii* has a wider distribution range from Mongolia to Japan and northern Indochina, but is not native to South Korea. Furthermore, the majority of sequenced *L. fischeri* samples (13 out of 16) exhibited considerable genetic divergence from the previously reported *L. fischeri* plastome (KT988070.1).

In this study, we analyzed two species and one subspecies, but genomic classification based on plastome and nrDNA was not feasible. In total, 106 SNPs were identified from whole-plastome sequences across 16 samples, which is comparable to the number of SNPs typically observed within a single species of plant genetic resources. According to previous studies, *Euonymus hamiltonianus,* a wild plant species, was reported to have 270 intraspecific SNPs [54], while *Peucedanum japonicum* showed 227 [55]. In the genus *Brassica*, 1,549–2,700 SNPs have been identified, with *Brassica napus* specifically showing 1,549 intraspecific SNPs [56–58]. Additionally, 3,592 interspecific SNPs have been reported in the genus *Oryza* [59]*.* This indicates that the genetic variation observed in the two species and subspecies analyzed in this study corresponds to the level of diversity typically found within a single species.

Furthermore, considering that interspecific hybridization occurs readily in nature or through artificial crosses, it may be more appropriate to treat these taxa as a single species. However, taxonomists argue that the three species exhibit clear morphological distinctions [23,24]. If this is indeed the case, it is possible that polyploidization events—either allopolyploid or autopolyploid—have occurred during their evolutionary history. Therefore, further studies integrating genome-wide SNP analyses and cytogenetic approaches are required to confirm putative interspecific hybridization and polyploidization events, and to clarify the taxonomic and evolutionary relationships among these taxa.

### Genetic Diversity of *L. fischeri* at High Altitudes of Mt. Hallasan

We focused on natural populations of *L. fischeri* inhabiting the Mt. Hallasan region, which harbors genetically diverse populations distributed across various altitudes. *L. fischeri* individuals collected from five different altitudes were sequenced and their plastomes were assembled to perform variant analysis (Fig 4D and 4E). Notably, *L. fischeri* individuals collected from 1400 m and 1905 m showed minimal differences in SNPs and InDels, whereas both populations exhibited clear divergence from those growing at intermediate altitudes, with 102 distinct SNPs identified between them. Phylogenetic analyses based on plastome and nrDNA sequences (Fig 4B and 4C) revealed differing topologies, similar to those observed in interspecific comparisons (Fig 3), suggesting interspecific hybridization. Similar patterns of nuclear-plastid phylogenetic incongruence have been reported in other Asteraceae genera, including Helianthus and Senecio, where introgression and chloroplast capture result in conflicting gene trees [60–62]. Furthermore, the plastome phylogenetic tree (Fig 2) shows a close relationship with species not native to the Korean Peninsula, such as *L. veitchiana* and *L. biceps*, supporting the possibility of historical introduction of these lineages. These findings highlight the complexity of evolutionary relationships within *L. fischeri* and emphasize the importance of integrating multiple genomic regions to accurately infer population structure and evolutionary history.

To further confirm this genetic diversity, we developed intraspecific SNP and InDel markers (Fig 5). These markers were applied to 72 samples, including 18 individuals of *L. fischeri*, 3 of *L. fischeri* var. *spiciformis*, 7 of *L. stenocephala* and 44 from farm collections (Fig 6). Although five genotypes were distinguished, clear species-level separation was difficult to achieve due to extensive genetic admixture. This likely reflects the breakdown of ecological isolation within the Korean Peninsula, either naturally or through human influence, enabling random interbreeding. We presumed that most populations of *L. fischeri*, *L. fischeri* var. *spiciformis*, and *L. stenocephala* already possess extensive genetic variations due to frequent hybridization. Similar organellar-marker studies have been conducted in other taxa [63–66]. However, many of these studies also note that plastome-based markers or low-density nuclear markers may lack sufficient resolution when *interspecific hybridization* or *polyploidization* events are involved, highlighting the limitations of our current approach. In addition, plastome heteroplasmy and concerted evolution of 45S nrDNA arrays may influence the observed sequence variation and phylogenetic inference. While these phenomena were not directly measured in this study, they represent inherent molecular limitations that should be considered when interpreting the results.

## Supporting information

S1 File Raw images. (PDF)

S2 File  S5 Table. (XLSX)
