# Supplementary material for: Genomic diversity of Ligularia revealed by complete plastid genomes and nuclear ribosomal DNAs from 16 collections in Korean Peninsula and Mt. Hallasan
Source: PLoS One. 2026 Feb 19;21(2):e0343215. doi: 10.1371/journal.pone.0343215 (PMC12919781; doi:10.1371/journal.pone.0343215)

Original uncropped gel images used in Fig 5.

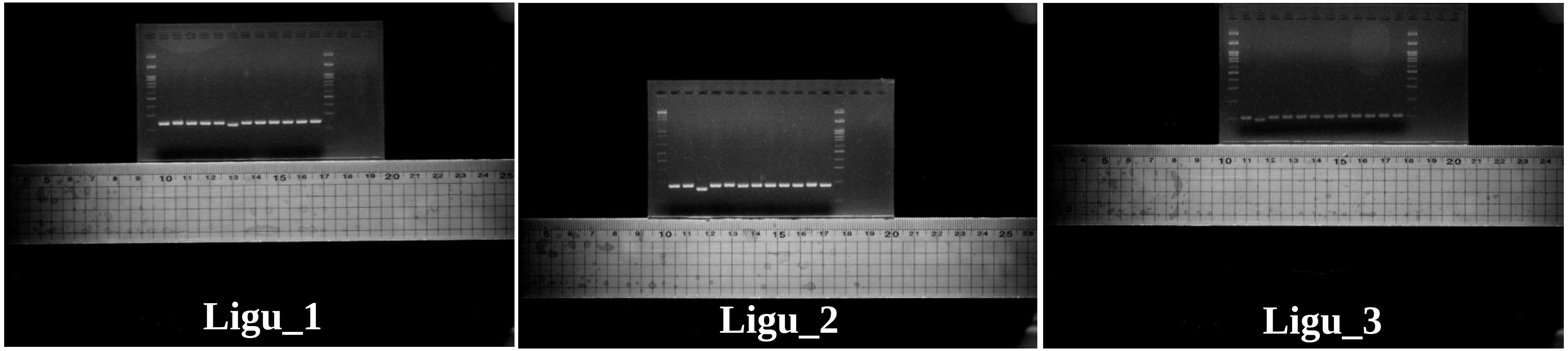

Original raw data underlying the genotyping results shown in Fig 6.

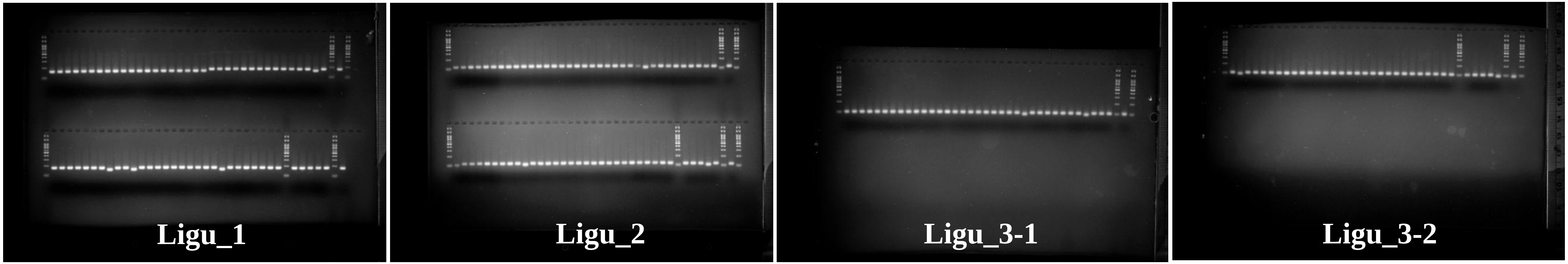

Supplement: S1 File — (PDF) [file pone.0343215.s001.pdf]
